# Robo3A and Robo3B expression is regulated via alternative promoters and mRNA stability

**DOI:** 10.1186/s12935-016-0347-9

**Published:** 2016-09-20

**Authors:** Anke Ruedel, Mandy Schott, Thomas Schubert, Anja Katrin Bosserhoff

**Affiliations:** 1 Institute of Biochemistry, Emil-Fischer-Zentrum, Friedrich-Alexander University of Erlangen-Nürnberg, Fahrstrasse 17, 91054 Erlangen, Germany; 2 Institute of Pathology, Friedrich-Alexander University of Erlangen-Nürnberg, Universitätsstrasse, 91054 Erlangen, Germany

**Keywords:** Repellent factor, Robo, Rheumatoid arthritis, Malignant melanoma

## Abstract

**Background:**

The transmembrane receptor family Roundabout (Robo) was described to have an essential role in the developing nervous system. Recent studies demonstrated that Robo3 shows an altered expression in rheumatoid arthritis as well as in melanoma.

**Context and purpose of the study:**

Until today no detailed studies of the two Robo3 isoforms (Robo3A and Robo3B) and their roles in rheumatoid arthritis synovial fibroblasts, respectively malignant melanoma are available. To get a better understanding regarding the role of Robo3A and Robo3B in the molecular process of rheumatoid arthritis and melanoma the exact characterization of expression and regulation is object of this study.

**Results:**

mRNA and protein expression of the transcriptional variants were analyzed by quantitative RT-PCR respectively western blotting and revealed particularly enhanced expression of Robo3B in rheumatoid arthritis and melanoma. Promoter assays and inhibitor studies also disclosed that there is apparently a cell- and isoform-specific regulation of the Robo3 expression. Finally, dissimilar mRNA stabilities of Robo3A and Robo3B are identified as decisive posttranscriptional gene expression control.

**Conclusion:**

In summary, this study supported an isotype specific role of Robo3B in disease hinting to different functional roles of each isoform.

## Background

The repellent factor family of Slit and their receptors Roundabout (Robo) were originally described to be involved in path-finding of commissural neurons [1]. In this context, Robo receptors act synergistically with other receptors and ligands to enable or prevent midline crossing of neurons. The Robo/Slit system is involved in regulating cell–cell and cell–matrix interactions of migrating cells during embryonic development, mediate cell adhesion of fibroblasts and induce tumor angiogenesis [2, 3].

There are four human Robo transmembrane receptors: Robo1/DUTT1, Robo2, Robo3/Rig-1 and Robo4/Magic Roundabout [4]. All share an extracellular domain consisting of immunoglobulin-like (Ig) and fibronectin type III motifs. The cytoplasmic domains vary between the Robo members [5, 6].

**Fig. 1:**
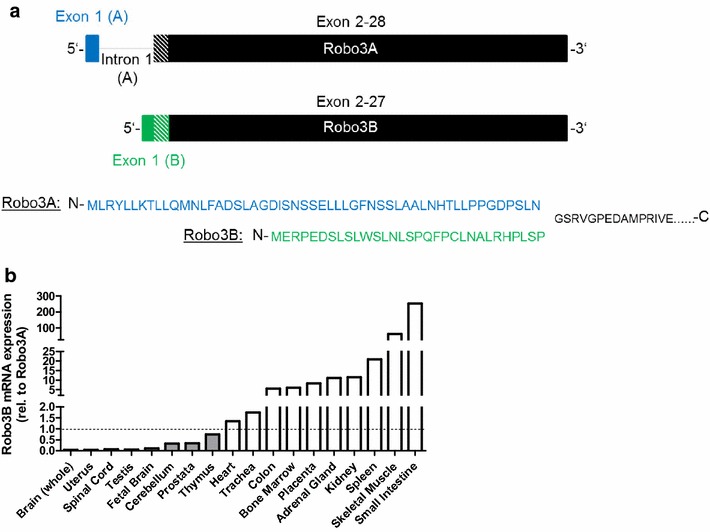
**a** Schematic structure of Robo3A and Robo3B. Robo3B lacks exon 1 of Robo3A and starts from an alternative transcription start site (TSS) in intron 1 of the NCBI reference gene, Robo3A [7]. The first 53 amino acids of Robo3A are not part of Robo3B. The Robo3B protein contains 33 amino acids, not being found in the Robo3A isoform (ENSEMBL). **b** Robo3B mRNA expression relative to Robo3A mRNA expression, determined via qRT-PCR. The majority of normal tissues exhibit a higher Robo3B expression compared to Robo3A, except all tissues of the brain and a few others

Recently, it became obvious that there are several splice variants of Robo receptors with distinct functions in defined developmental stages or tissues and that they are conserved between different species [7, 8]. The two Robo3 isoforms, Robo3A and Robo3B described by Camurri et al. differ only by a few amino acids at the N-terminus [7]. The variant Robo3B lacks exon 1 of Robo3A and starts from an alternative transcription start site (TSS) in intron 1 of the NCBI reference gene (Fig. 1a). The first 53 amino acids of Robo3A are not part of Robo3B. The Robo3B protein contains 33 amino acids, not found in the Robo3A isoform (ENSEMBL) (Fig. 1b). Whereas the longest variant of Robo3, Robo3A, is incapable to bind the ligand Slit, the shorter variant, Robo3B, is able to bind Slit [1, 7]. So far, the expression levels of the two Robo3 variants are not determined in different cell types and disease-related changes of expression are not addressed.

Previous studies of our group detected significantly increased Robo3 expression in rheumatoid arthritis synovial fibroblasts (RASF) compared to SF and in melanoma cell lines compared to melanocytes (NHEM) [9, 10]. This finding hints to deregulation of Robo3B in disease, however, no details about the specific isoforms, Robo3A and Robo3B, exist.

To get a better understanding regarding the role of the two Robo3 isoforms, the expression in different cell types as well as in RASF and different melanoma cell lines were analyzed at first. Promoter assays, inhibitory studies and mRNA stability experiments were used to explain the results obtained from the different expression pattern of Robo3A and Robo3B.

## Methods

### Cell lines and tissue culture

Synovial tissue samples from patients with RA were obtained immediately after opening the knee joint capsule as previously described [9, 11, 12]. RASF were cultured in DMEM (Sigma-Aldrich, St. Louis, Missouri, USA) + 10 % fetal calf serum (PAN Biotech, Aidenbach, Germany) + penicillin/streptavidin (PAN Biotech). These cells were incubated under humid conditions in a 5 % CO_2_ incubator at 37 °C.

The human melanoma cell lines Mel Im, A375, Hmb2, HTZ19d, Mel Wei, Mel Ei were maintained in DMEM and Mel Juso in RPMI (Sigma-Aldrich) supplemented with 10 % FCS and P/S. The cells were cultured in an incubator at 37 °C with 8 % CO_2_.

### Isolation of RNA, reverse transcription and quantitative real-time PCR

Total cellular RNA was isolated from cultured cells using the Total RNA Kit (VWR, Darmstadt, Germany) and cDNAs were generated as described before [13]. Real-time quantitative PCR (qRT-PCR) was performed using SYBR Green master mix (QIAGEN) with primer pairs for Robo3A (5′- GAACTTGTTCGCGGACTCTC -3′ and 5′- CCCGTTCTTGTACCACTCA -3′), Robo3B (5′-CCCTCTGGAGCCTCAATCTC-3′ and 5′-CCCCGTTCTTGTACCACTCA -3′), β-actin (5′-CTACGTGGCCCTGGACTTCGAGC-3′ and 5′-GATGGAGCCGCCGATCCACACGG-3′) and MIA (5′-TTCAGGGTGACTACTACGGTCGCCTGGCTGCTCGTCTGGG-3′ and 5′-CCCAGACGAGCAGCCAGGCGACCGTAGTAGTCACCCTGAA-3′). Relative gene expression was normalized to β-actin mRNA levels using the comparative cycle threshold (Ct) method. Specificity of the Robo3 isoform primers was confirmed by sequencing of the PCR products, Robo3A and Robo3B.

### Protein isolation and western blot analysis

Protein extracts from primary cells and cell lines were homogenized in 100 or 200 μl RIPA-buffer (50 mM Tris–HCl pH 7.5; 150 mM NaCl; 1 % (w/v) Nonidet^®^ P40; 0.5 % (w/v) Natriumdesoxycholat; 0.1 % (w/v) SDS; Protease inhibitors). Insoluble fragments were removed by centrifugation at 13,000 rpm for 10 min. The supernatant was stored at −20 °C. For western blot, protein lysates were separated on 7.5 % SDS-PAGE gels and blotted onto a PVDF membrane [14]. After blocking with 5 % milk powder/TBS-T (1 %), primary antibodies were applied (anti-Robo3B, 1:500, BioGenes; anti-β-actin, 1:4000, Sigma). Alkaline phosphatase-conjugated anti-rabbit (1:4000, cell signaling) or anti-mouse antibody (1:3000, NEB) served as secondary antibodies.

### Peptide-antibody production

A specific antibody was generated against the amino acids 18-31 of Robo3B (QFPCLNALRHPLSP) in rabbits by BioGenes GmbH (Berlin).

### Cloning of Robo3A and Robo3B promoter region

Human Robo3 from −500 to −1 bp relative to the translation start was amplified by PCR (Robo3A: 5′-GACGGTACCGGGCAGAAGG-3′ and 5′-GACAAGCTTCTGCAGCAGCGTTTTC-3′; Robo3B: 5′- CCCTTGAAATGAAGCGTGATTATCC-3′ and 5′-CTCCTATGCTTCTCTGCGGAGC -3′) using Taq^®^-Polymerase (Roche, Mannheim, Germany). Subsequently, the amplified fragments were cloned into a pGL4basic vector (Promega Corporation, Madison, WI, USA) via HindIII-HF (NEB, Frankfurt am Main, Germany) and KpnI-HF (Robo3A) respectively EcoRV-HF (Robo3B) restriction sites. The vector sequences were confirmed by Sanger sequencing.

### Reporter gene assays

The tumor cell lines (150,000–200,000 cells) were cultured in six-well plates for 24 h and then treated with cationic lipid/plasmid DNA suspension: 1 µg of luciferase reporter plasmid (Robo3A, Robo3B or an empty pGL4basic vector) and 0.1 µg of the internal control plasmid pRL-TK. Twenty-four hours after the transfection the cells were harvested and the lysate was analyzed for luciferase activity with a luminometer using Promega dual-luciferase assay reagent [14]. At least three independent transfection experiments were performed for each construct.

### Cell treatments

The melanoma cell line Mel Ei (150,000 cells) were cultured in six-well plates and were treated for 24 h with different inhibitors (SB431542 InvivoGen 2 μM; S3I-201 Sigma-Aldrich 20 mM; Dorsomorphin Tebi-bio 2 mM; LY-294002 Sigma-Aldrich 20 mM; Wortmannin Sigma-Aldrich 10 mM; UO126 Calbiochem 10 mM; Vemurafenib Active Biochem 100 μM; KT5720 Merck Millipore 100 nM; Bisindolylmaleimide II Santa Cruz 10 μM; Gö 6983 Santa Cruz 10 μM; Wnt Agonist Calbiochem 10 μM). For the stability assay the cells were treated with α-Amanitin (AppliChem, 5 mM). After isolation of total RNA, quantitative RT-PCR was performed to detect relative gene expression. Normalization was based on mRNA input in this experiment.

### Statistical analysis

Calculations were performed using the GraphPad Prism software (GraphPad software, Inc., San Diego, USA). All results are indicated as mean ± SEM and comparison between groups were made using the Student paired *t* test (*: *p* < 0.05; **: *p* < 0.01; ***: *p* < 0.001; ns: not significant).

## Results

### Robo3 expression in normal tissues

It is already known that the receptor Robo3 is ubiquitous expressed in all normal tissues (see proteinatlas.org). However, no details concerning the expression of the two isoforms (Fig. 1a) are existent, as all previously used antibodies recognize both variants and primers used in RT-PCR by most studies do not differentiate both variants. Due to the lack of available data concerning the expression pattern of the Robo3 isoforms, the mRNA expression levels of Robo3A and Robo3B were determined in several tissues. Two mRNA tissue banks (Total RNA, 20 human Tissues, Clontech Laborities, Inc., Mountain View, California, USA; total RNA, human tissues, Ambion, Kaufungen, Germany) were used which contain samples from various tissues. After generating cDNA, the expression of Robo3A and 3B was analyzed via qRT-PCR. The results showed commonly a higher expression of Robo3B compared to Robo3A given as the ratio (Fig. 1b). In almost all mesenchymal tissues Robo3A expression is low and in some tissues not detectable at all (Table 1). The higher Robo3A level in all tissues of the brain is remarkable.

**Table 1:** mRNA expression of Robo3A and Robo3B relative to β-actin (via qRT-PCR)

Tissue	Robo3A	Robo3B
Fetal liver	Not detectable	1.17E−05
Lung	Not detectable	1.13E−05
Liver	Not detectable	3.71E−06
Salivary gland	Not detectable	3.01E−06
Adrenal	Not detectable	6.89E−07

### Robo3 expression in RASF compared with normal SF

**Fig. 2:**
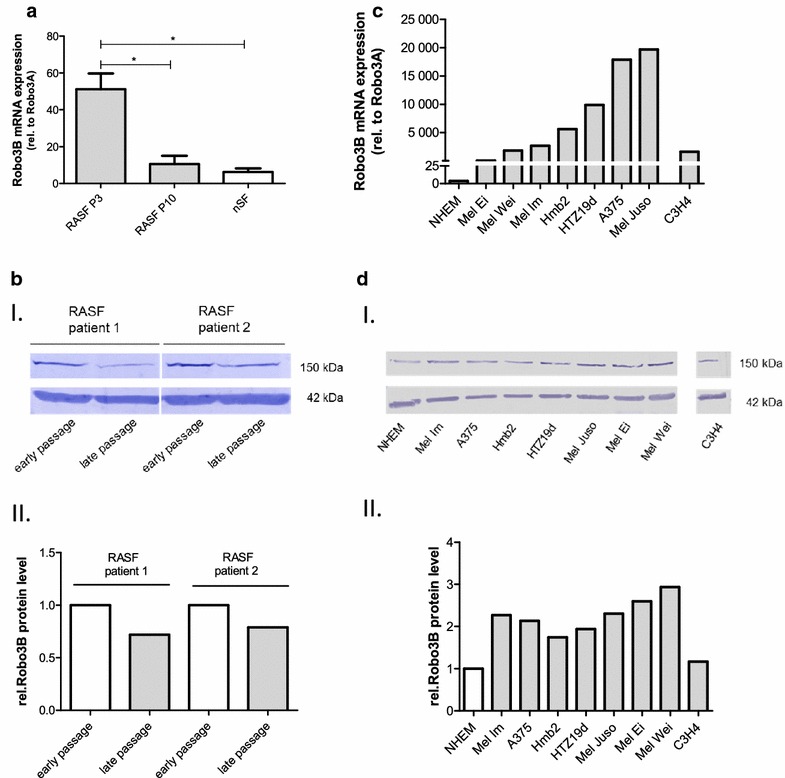
Robo expression in RASF as well as in melanoma cell lines. **a** mRNA levels of the two Robo3 isoforms in RASF in early and late passages using specific primers for both transcription variants. **b** Western blot analysis using the designed Robo3B-specific antibody (I.) showing the 150 kDa Robo3B protein (β-actin as reference, 42 kDa) and densitometric analysis normalized by β-actin (II.). **c** Robo3B mRNA expression relative to Robo3A in melanoma cell lines as well as in neuroglioma cell line (C3H4). **d** Protein expression of the tumor cell lines analyzed by the Robo3B-specific antibody (I.) and densitometric analysis normalized by β-actin (II.)

Recently, a strong induction of Robo3 expression in activated RASF compared to expression in normal synovial cells was determined [9]. To examine the different expression of the two Robo3 isoforms in RA, the level of mRNA of Robo3A and Robo3B was determined in RASF in early and late passages based on the previous study. Robo3A mRNA expression was very low in RASF, whereas Robo3B mRNA showed enhanced expression in early versus late passages RASF (Fig. 2a). Thus, all tested RASF displayed a higher expression of Robo3B than Robo3A. The overall expression of the two Robo3 isoforms in normal SF from healthy donors was found to be at a similar level in RASF in late passages (data not shown). For detection of protein levels of Robo3B a Robo3B-specific polyclonal antibody directed against the N-terminus of Robo3B was generated as commercially available antibodies did not recognize isoform-specific regions. Western blot analysis using this Robo3B antibody confirmed the elevated expression of Robo3B in RASF in early passages on protein level, whereas expression was reduced in later passages, clearly correlating with mRNA expression (Fig. 2b).

### Robo3 expression in tumor cell lines compared to melanocytes

To investigate if tumor cells show different expression patterns of Robo3A and Robo3B tumor cells were analyzed, starting with melanoma. Here, mRNA levels of the two Robo3 isoforms were determined in different melanoma cell lines. Robo3A expression was very low, whereas Robo3B showed enhanced expression when comparing the ratio (Fig. 2c). Western blot analysis with the Robo3B-specific antibody also showed a stronger Robo3B protein expression compared to melanocytes (Fig. 2d). Due to the important role of Robo3 in the developing nervous system, a neuroglioma cell line (C3H4) was analyzed and also showed strong Robo3B expression (mRNA and protein). The protein expression of C3H4 is lower compared to the melanoma cell lines, but stronger in relation to primary melanocytes.

### Regulation of the Robo3 isoforms through transcription factors

**Fig. 3:**
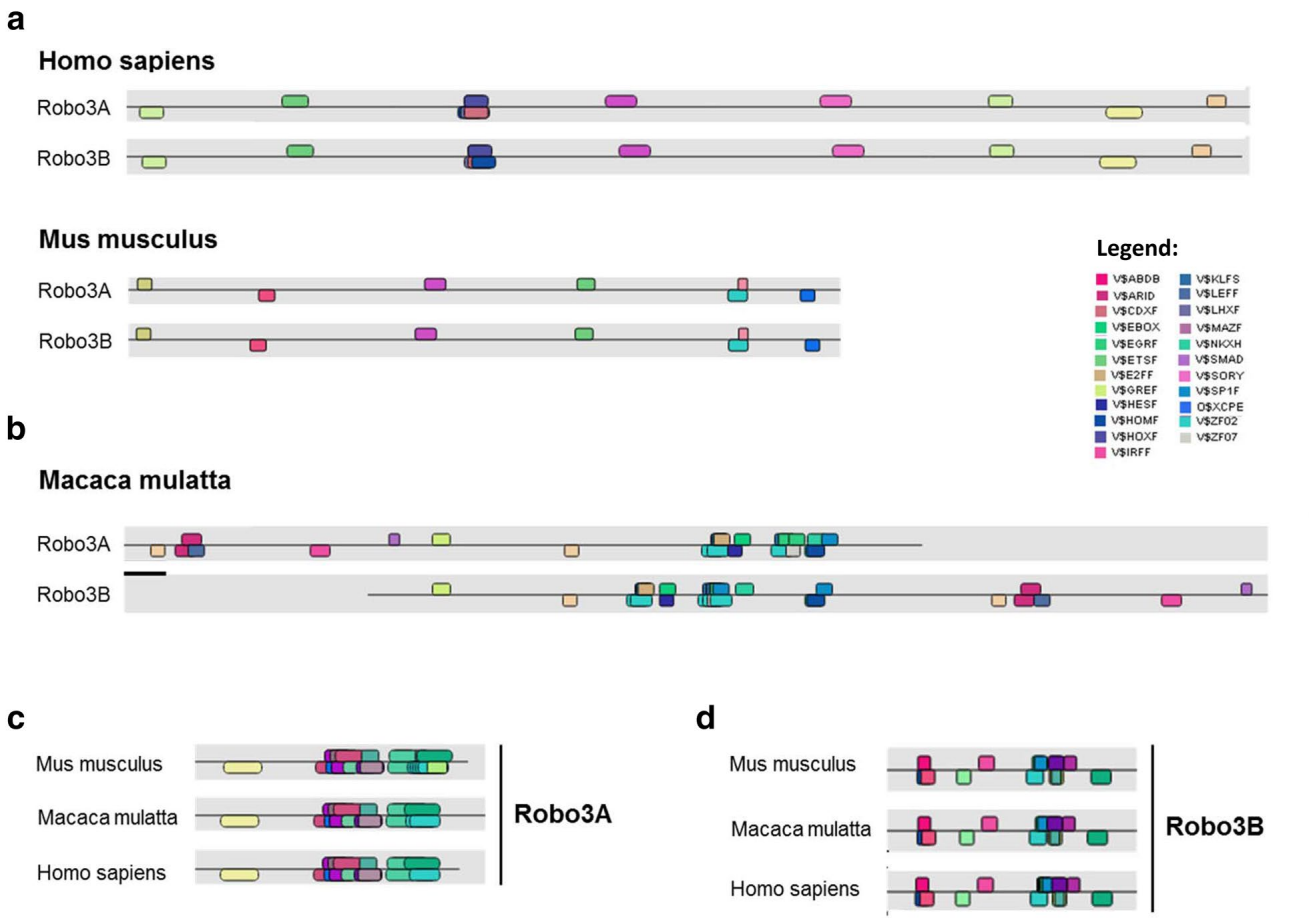
Promoter analysis using MatInspector Software (Genomatix Software GmbH, Munich). **a** The predicted promoter regions of Robo3A and Robo3B revealed similar clusters of transcription factor binding sites (TFBS) in *Homo sapiens* and *Mus musculus* whereas the nucleotide sequences differ completely. The *Robo3B* promoter sequence of *Macaca mulatta* was analyzed analogously to the human and mouse promoter sequences of Robo3B and also revealed different promoter sequences of *Robo3A* and *Robo3B* but matching TFBS clusters. **b** Several of the TFBS clusters were conserved between the species. **c**, **d** Comparison of the promoter regions between the species showed conserved TFBS patterns for Robo3A (**c**) and for Robo3B (**d**)

Analyses of the predicted promoter regions of *Robo3A* and *Robo3B* using the MatInspector Software tool (Genomatix Software GmbH, Munich) revealed completely different nucleotide sequences but interestingly similar clusters of transcription factor binding sites (TFBS) in *Homo sapiens* and *Mus musculus* (Fig. 3a). Because of this result, the *Robo3* promoter sequences of *Macaca mulatta* were analyzed as well. Again, these revealed different promoter sequences of Robo3A and Robo3B but matching TFBS clusters (Fig. 3b). Furthermore, several clusters are conserved between the species. Comparison of the promoter regions between the species showed conserved and discriminating TFBS patterns for Robo3A (Fig. 3c) as well as for Robo3B (Fig. 3d). This involves for example zinc finger (ZF02), E-box binding factors (EBOX) and LEF1/TCF, to name a few transcription factor families. There are, however, also differentiating transcription factors such as STAT5 (signal transducer and activator of transcription 5) and SMAD4 (Smad4 transcription factor involved in TGF-beta signaling), which binding sequences occurred just in the predicted promoter region of Robo3B.

**Fig. 4:**
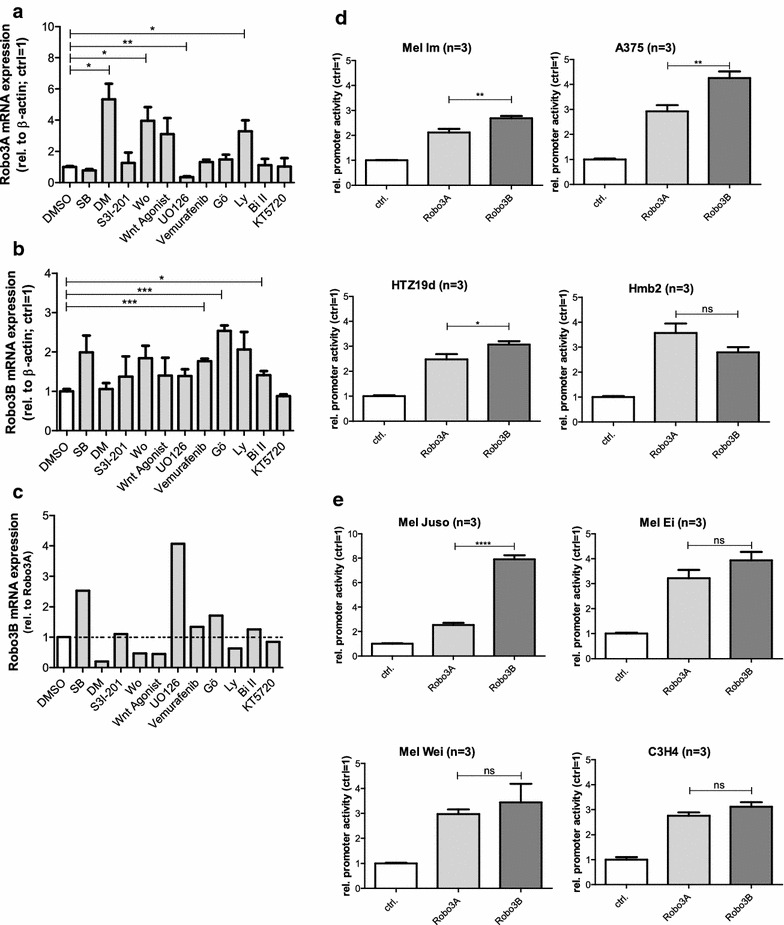
Regulation of the two Robo3 isoforms. **a**–**c** Mel Ei were treated with several inhibitors for 24 h. The amount of Robo3A (A) respectively Robo3B (B) mRNA expression was quantified by real-time (RT)-PCR and showed varied expression pattern especially when comparing the ratio of Robo3B relative to Robo3A (C). **d, e** Reporter gene assays using specific Robo3A or the Robo3B promoter plasmid. Luciferase activity was measured in melanoma cell lines as well as in neuroglioma cell line (C3H4). **d** A significantly increased promotor activity of the Robo3B promotor compared to the Robo3A promotor was measured in all metastasis cell lines of melanoma (Mel Im, A375, HTZ19d) except Hmb2. **e** No significant differences were found in primary tumor of melanoma except Mel Juso. Also no significant differences in the neuroglioma cell line

The result of the in silico analysis indicates different TFs, whose participation in cancer-associated pathways are already known and could also play a role in the regulation of Robo3 isoform expression. Therefore, an inhibitor screening was carrying out to examine potential transcription factors involved in the regulation of the two Robo3 isoforms. Whereas Robo3A expression is apparently regulated through PI3-kinase (significant expression differences after Ly and Wortmannin treatment), Robo3B expression is regulated by a serin/threonine protein kinase (significant expression differences after Gö6983, Bisindolylmaleimide II and Vemurafenib) (Fig. 4a, b). Especially when comparing the ratio of the expression changes of Robo3A and Robo3B after inhibitor treatment (Fig. 4c), these assays suggest the distinctive regulation of each Robo3 isoform.

### Analyses of Robo3A and Robo3B promoter activities

Based on the expression pattern of Robo3A and Robo3B in the melanoma cell lines, the regulation of the different promoters of the two isoforms was analyzed. Reporter gene plasmids containing either the Robo3A or the Robo3B promoter (−500 to −1 bp relative to translation start) were designed, cloned, transfected and luciferase activity assay were performed. The assays showed a tendency for a higher promotor activity of Robo3B compared to Robo3A in the tumor cell lines from melanoma metastasis except for Hmb2 with nearly similar activities of the promoter of both isoforms (Fig. 4d). The cell lines of primary malignant melanoma showed no significant differences between the activities of Robo3A and Robo3B promoter except Mel Juso with significant higher Robo3B promoter activity (Fig. 4e). Also in the neuroglioma cell lines no difference in activity of the promoters could be measured. These results do not explain the higher Robo3B expression on mRNA or protein level.

### Stability assay

**Fig. 5:**
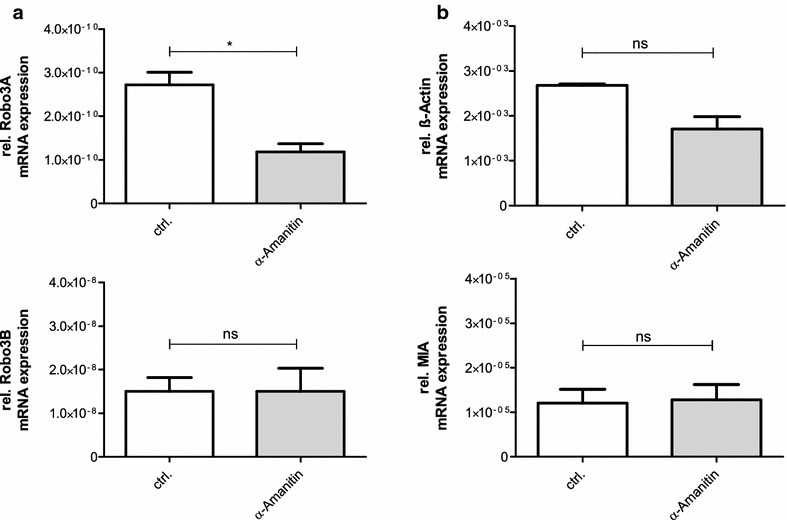
Mel Ei were treated with the transcriptional inhibitor a-amanitin for 24 h. **a** mRNA expression levels of the two Robo3 isoforms determined via qRT-PCR show a significant decrease of the relative Robo3A expression after 24 h, whereas the relative Robo3B expression is nearly unchanged. **b** Two reference genes (MIA and β-actin) with already known differing mRNA stabilities are used. No significant variances were measured

To determine the cause of the different expression pattern of Robo3A and Robo3B other possible regulatory mechanisms were analyzed. In addition to the control of mRNA level by transcription factors, the regulation of mRNA turnover, is very important [15]. The treatment with α-amanitin, a RNA polymerase II and III inhibitor, revealed a significant decrease of the Robo3A mRNA expression whereas the Robo3B mRNA expression is nearly stable after 24 h (Fig. 5a). Therefore, the cell line Mel Ei with different Robo3A and Robo3B mRNA expression but similar promoter activities was used. (For other cell lines this assay was not feasible due to low expression of Robo3A). The results were shown as raw data relating to two reference genes (β-actin and MIA) which clarifies that the α-amanitin treatment has no effect of the expression of the two reference genes that serve as control after 24 h (Fig. 5b).

## Discussion and conclusion

In the last few decades deregulation of the Robo/Slit system can be linked to several diseases. Especially altered expression in several cancer types has been described in recent studies [16]. However, not much is known about the role of the two transcriptional variants of Robo3, which were described already in 2005 [7]. Interestingly, studies revealed that variant A is incapable of ligand binding and therefore seems to have a different function. However, all recent studies focusing on Robo3 ignored this fact including our own studies and used tools, which do not distinguish the variants [9, 17, 18].

Previously, our group revealed that Robo3 expression is altered in synovial fibroblasts of rheumatoid arthritis patients [9]. Currently, there are no commercially available Robo3A- or Robo3B-specific antibodies. After generating of a Robo3B-specific antibody, it was possible to show that Robo3B is the up-regulated isoform in RASF and melanoma at mRNA and protein level.

By analyzing the expression pattern of the Robo3 isoforms in several tissues, it was observed that Robo3B is the predominant isoform. Based on the fact of the high expression of Robo3B in mostly all mesenchymal tissues it can be speculated that this isoform might play an increasingly crucial role in comparison to Robo3A. Furthermore, the enhanced Robo3B expression in RASF and melanoma is an indication that the upregulation contribute to tumorigenesis and progression. Denk et al. already showed a connection between the deregulation of Robo3 in RASF and the aggressiveness of the fibroblasts [9]. The increased Robo3 expression in early passages but decreased expression in later passages suggests the involvement in the initial stage of RA, especial in the migration and invasion of SF in RA. The results of this new study indicate that the variant B plays the crucial role for the enhanced migration potential of RASF and melanoma cells. In this context, the predominant Robo3B expression in mostly all mesenchymal tissues and the upregulation in diseases indicate that these cells apparently undergo epithelial to mesenchymal transition (EMT) [19]. In addition, a recent study demonstrated that Robo3 over-expression promotes growth, invasion and metastasis of pancreatic cancer cells [17]. All these facts advocate this hypothesis.

Interestingly, the higher Robo3A expression compared to Robo3B in several neuronal tissues might be an indication that Robo3A has a specific function in the brain. It is already known that the transmembrane receptor Robo functions as axon guidance molecule and that mutations in the Robo3 gene can lead to an autosomal recessive disorder [20]. The observed up-regulated Robo3B mRNA expression in the neuroglioma cell line also suggests that the deregulation of Robo3B can be connected to tumor development of the central nervous system. Summarizing the expression pattern in healthy and diseased tissue on mRNA and protein level, a clear cell-type dependent regulation of Robo3A and Robo3B was observed.

In order to understand the underlying regulation of expression possible signaling pathways for the control of gene expression were investigated. The differential expression of the Robo3 isoforms could to be due to the usage of alternative promoters. About 30–50 % of the human genes are under the control of alternative promoters [21, 22]. In this case, the expression pattern of transcription factors in different cell types would be the reason for the distinct expression of the two isoforms. The gene expression control has already been described for the Robo1 isoforms. Here, alternative promoters and transcription start sites are used [23]. Nonetheless, the reporter gene assays performed in this study revealed significant differences in promoter activity only for some cancer cell lines. However, the results of the inhibitor assay suggest that the variants are regulated through different signaling pathways. Because not only one specific inhibitor showed significant expression changes it could be inferred that more than one specific pathway plays a role for the Robo3 isoforms. Particularly for Robo3B a serine/threonine kinase seems to be involved in the regulation, like already described for rheumatoid arthritis and melanoma [24, 25]. More than this, the significant expression change of Robo3B after treatment with Vemurafenib, a selective inhibitor of mutated BRAF, can be linked with malignant melanoma [26]. The inhibitor screening indicates a cell- and isoform-specific regulation nevertheless this does not completely correlate with the mRNA expression levels. Therefore, the expression of the isoforms cannot solely be regulated by alternative promoters but also has to be modified via additional mechanisms.

Since the previous experiments did not reveal a complete explanation, alternative regulation mechanisms on post-transcriptional or -translational levels, as speculated for Robo proteins in growing axons, were considered [27, 28]. The mRNA turnover is a complex system including the process of mRNA degradation with cis-acting and trans-acting elements [15]. In this study we demonstrated that the Robo3B mRNA is more stable than the Robo3A mRNA. Short half-lives are already known for many proto-oncogenes like *c*-*fos* and *c*-*myc* because of AU-rich elements in the 3′UTR [29, 30]. The most cis-acting elements are in the 3′UTR but such elements can also be found in the 5′UTR. For example, the interleukin-2 mRNA has a JNK response element (JRE) in the 5′UTR [31]. The specific binding of two RNA-binding proteins, recognize the 5′JRE, ensures the mRNA stability. Possibly, the Robo3B mRNA has such elements, which contribute to the stabilization. Also conceivable is the inactivation or loss of such elements in the Robo3A mRNA. Whatever the crucial regulatory element might be it has to be located in the 5′UTR because Robo3A and Robo3B differ only by a few amino acids at the N-terminus. In this respect, the differing 5′UTR lengths may lead to a remarkably divergent translational potential.

Taken together, this is the first study specifically analyzing expression of the two isoforms of the repellent factor Robo3. The results endorse that Robo3A and Robo3B are using alternative promoters whereby the regulation is apparently cell- and isoform-specific. Furthermore, also the mRNA stability seems to be crucial for the regulation. Especially Robo3B, which is able to bind the ligand Slit, apparently plays a substantial role in diseases and might be therefore a novel therapeutic target. Here, differentiation between both isoform is of strong importance.

## Abbreviations

Robo: roundabout
TSS: transcription start site
RASF: rheumatoid arthritis synovial fibroblasts
NHEM: normal human epidermal melanocytes
qRT-PCR: real-time quantitative PCR
TFBS: transcription factor binding sites
STAT5: signal transducer and activator of transcription 5
SMAD4: Smad4 transcription factor involved in TGF-beta signaling
TF: transcription factor
